# Modulation of Host Angiogenesis as a Microbial Survival Strategy and Therapeutic Target

**DOI:** 10.1371/journal.ppat.1005479

**Published:** 2016-04-14

**Authors:** Nir Osherov, Ronen Ben-Ami

**Affiliations:** 1 Department of Clinical Microbiology and Immunology, Sackler School of Medicine, Tel-Aviv University, Tel-Aviv, Israel; 2 Infectious Disease Unit, Tel-Aviv Sourasky Medical Center, Tel-Aviv, Israel; 3 Department of Medicine, Sackler School of Medicine, Tel-Aviv University, Tel-Aviv, Israel; McGill University, CANADA

## Angiogenesis in Health and Disease

The blood vascular system is essential to the development and maintenance of tissues of multicellular eukaryotes. Its roles include internal transport and delivery of oxygen and nutrients and immune surveillance and trafficking of cells and molecules of the innate immune system to sites of tissue damage. Formation of blood vessels occurs by two principal modes: assembly of endothelial progenitor cells into vascular networks (vasculogenesis), which takes place predominantly in embryonic life, and the expansion of existing vascular systems by sprouting of new blood vessels from existing ones (angiogenesis) [1]. Angiogenesis is a sequential process guided by angiogenic cues, notably, hypoxia inducible factor (HIF)-1, vascular endothelial growth factor (VEGF), fibroblast growth factor (FGF), angiopoietin-2, and chemokines released by hypoxic, inflammatory, or neoplastic cells [1].

Whereas physiological angiogenesis is essential for normal tissue growth, remodeling, and regeneration, dysregulated angiogenesis plays a pivotal role in disease states, including cancer, inflammatory diseases, atherosclerosis, and diabetic retinopathy [2]. Importantly, cancer cells can exploit angiogenesis to support their own proliferation and metastatic dissemination. This so-called tumor angiogenesis has been the focus of intense research, leading to the discovery of a novel class of antineoplastic drugs [3]. During infection, angiogenesis is induced when microbial motifs are detected in concert with damage-associated molecular patterns. Specifically, bacterial ligands such as LPS and unmethylated CpG activate mammalian Toll-like receptors (TLRs) 2, 4, 7, and 9, while adenosine, a danger signal that accumulates rapidly in ischemic or damaged tissues, synergizes with TLRs to induce the synthesis and release of VEGF and recruitment of endothelial progenitor cells [4]. The ensuing inflammatory angiogenic response facilitates the migration of leukocytes to infected tissue and wound repair. Moreover, an emerging concept links angiogenesis to innate immunity, implying that an adequate angiogenic response is required for control and clearance of invading pathogens [5–7]. Intriguingly, some microbial pathogens manipulate the host angiogenic response, either suppressing it to enhance their persistence in tissues or hijacking angiogenesis for their own ends. Deciphering such interactions may uncover new therapeutic targets for some of the most tenacious infectious diseases. In this mini-review, we highlight examples where modulation of host angiogenesis has been shown to play an important role in microbial pathogenesis.

## Regional Events at Infection Sites Control Microbial Sequestration and Killing

The evolution of a circulatory system enables a systemic immune response but opens the way for rapid dissemination of pathogens within the host. Rapid microbial dissemination is controlled via early local events that wall off invading pathogens [8]. Microbial sequestration addresses contrasting needs; it must enable migration of immune cells and antimicrobial molecules into infected tissue while preventing pathogens from gaining access to the circulatory system. Failure to achieve these goals results in microbial persistence or dissemination, respectively. The early events that occur within hours of microbial invasion include triggering of the complement cascade and platelet aggregation followed by the expression of adhesion molecules (endothelial-leukocyte adhesion molecule [ELAM]-1, intercellular adhesion molecule [ICAM]-1, and vascular cell adhesion molecule [VCAM]-1) on activated endothelial cells, facilitating the influx of immune cells to infected tissue [9].

The microenvironmental conditions at the site of infection are characterized by low oxygen pressure and high concentrations of lactate and reductive metabolites. This is especially true if the local vasculature is directly disrupted by infection. The heterodimeric transcription factor HIF-1 is the pivotal regulator of angiogenesis and myeloid cell function under hypoxic conditions. HIF-1α levels are dynamically controlled by oxygen-dependent prolyl hydroxylase domain (PHD) proteins that regulate HIF stability [10]. Moreover, HIF-1 and NF-kB signaling are strongly interdependent, with intact NF-kB signaling shown to be required for hypoxic HIF-1 induction [11,12]. HIF-1 activation is observed in infections with bacteria, viruses, fungi, and protozoa [13]. Interestingly, hypoxia-independent activation of HIF-1α is induced by iron deprivation, suggesting that bacterial siderophores may also trigger this pathway [14]. Myeloid aggregation, motility, invasion, and bacterial killing are critically dependent on HIF-1α, which allows myeloid cells to function under conditions of low oxygen pressure by switching to glycolytic metabolism [15]. In sum, HIF-1 activation of VEGF signaling and angiogenesis likely act in concert with myeloid cell activation and trafficking to keep tissue-invasive pathogens in check.

## Some Pathogens Enhance Host Angiogenesis to Support Infection

Infection-associated angiogenesis has been described in diverse infections caused by bacteria, viruses, protozoa, and fungi (Table 1). Conceptually, infectious angiogenesis may be classified as either direct induction of host angiogenesis by pathogen-derived molecules or angiogenesis driven by a nonspecific host inflammatory response. Both *Bartonella henselae* and Kaposi sarcoma-associated herpesvirus (KSHV) induce rampant angiogenesis, resulting in severe illness in persons with deficient cellular immunity, such as patients with AIDS. The *B*. *henselae* adhesin A (BadA) and type IV secretion system VirB/D4 mediate bacterial endothelial cell adherence and uptake followed by activation of a proangiogenic phenotype, thereby expanding the host cell habitat of this pathogen [16]. KSHV expresses several factors that either directly activate the formation of blood vessels (viral interleukin 6 [vIL-6], vCCL-1, and vCCL-II) or indirectly activate cell pathways, leading to angiogenesis (vGPCR, vFLIP, K1, K15, KSHV miRNAs) [17]. Virus-driven angiogenesis enables propagation of KSHV by recruiting uninfected endothelial lineage and hematopoietic cells for further infection and reactivation of KSHV in latently infected cells [18].

**Table 1:** Notable pathogens associated with modulation of host angiogenesis.

Pathogens associated with pro-angiogenesis	Mechanisms discovered	References
*Bartonella henselae*	Reprogramming of human myeloid cells towards a tumor-associated macrophage–like proangiogenic phenotype.	[32]
	*Bartonella* adhesin A (BadA) mediates binding to fibronectin, adherence to endothelial cells, and secretion of VEGF.	[16]
	The type IV secretion system VirB/D4 translocates several *Bartonella* effector proteins (Beps) into the cytoplasm of infected endothelial cells, resulting in uptake of bacterial aggregates, inhibition of apoptosis, and activation of a proangiogenic phenotype.	[33]
*Mycobacterium tuberculosis*	Mycobacteria induce abnormal leaky granuloma-associated angiogenesis, which promotes mycobacterial growth and increases spread of infection to new tissue sites.	[6,19]
*Candida albicans*	*C*. *albicans* stimulates vascularization in infected brain and kidney abscesses and activates endothelial cell genes involved in chemotaxis and angiogenesis.	[34,35]
Kaposi Sarcoma Herpesvirus (KSHV)	KSHV expresses molecules that directly activate the formation of blood vessels: viral interleukin 6 (vIL-6), vCCL-1, vCCL-II, vGPCR, vFLIP, K1, K15, and KSHV miRNAs.	[17,18]
Cytomegalovirus (CMV)	CMV-secreted pUL7 carcinoembryonic antigen-related cell adhesion molecule (CEACAM)–related protein induces angiogenesis in endothelial cells via STAT3/ERK1/2 activation and IL-6 secretion.	[36]
Hepatitis C virus (HCV)	HCV-mediates hepatic angiogenesis by stabilizing cellular HIF-1α via the NF-κB pathway to up-regulate VEGF and other proangiogenic factors.	[37]
Human papillomavirus (HPV)	HPV E6 protein inhibits p53 and stabilizes HIF-1α to up-regulate VEGF, favoring formation of new blood vessels and increasing permeability of existing blood vessels.	[38]
*Schistosoma mansonii*	*S*. *mansonii* soluble egg metabolites induce hepatic neovascularization by up-regulating endothelial cell VEGF as well as directly inducing endothelial cell proliferation, migration, and sprouting.	[39–41]
**Pathogens associated with inhibition of angiogenesis**		
*Bacillus anthracis*	*Bacillus anthracis* protective antigen (PA) inhibits VEGF and basic fibroblast growth factor (bFGF)-induced endothelial cell angiogenesis.	[42]
*Pseudomonas aeruginosa*	*P*. *aeruginosa* hemolytic phospholipase C at picomolar concentrations is selectively lethal to endothelial cells and inhibits angiogenesis.	[43]
*Aspergillus fumigatus*	Down-regulation of HIF-1α, VEGF-A, bFGF, and VEGF receptors 1 and 2 is dependent on *A*. *fumigatus* secondary metabolism under the transcriptional regulation of *LaeA*.	[7,24]

**Fig 1:**
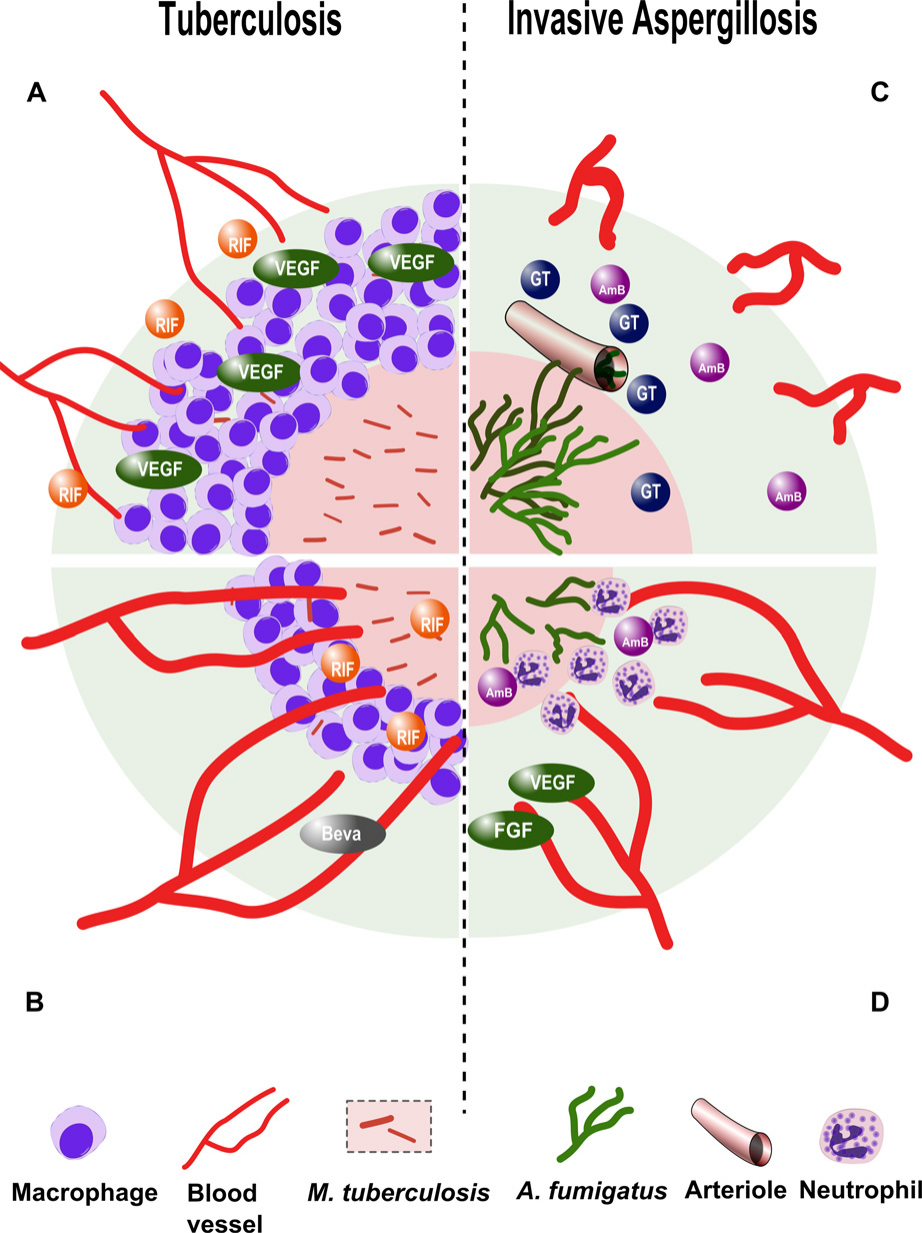
Modulation of angiogenesis in tuberculosis and invasive aspergillosis. A. Vascular endothelial growth factor (VEGF)-mediated, host-induced pathological angiogenesis in *M*. *tuberculosis* granulomas restricts perfusion of the granuloma core and attenuates antituberculosis drug efficacy of rifampicin (RIF). B. Treatment with the angiogenesis-inhibiting drug bevacizumab (Beva) reverses pathological angiogenesis, enhances perfusion of the granuloma core, and synergizes with rifampicin. C. *A*. *fumigatus* hyphae invade pulmonary arterioles and induce intravascular thrombosis. The compensatory angiogenic response is down-regulated by gliotoxin (GT) and other fungal secondary metabolites, further limiting perfusion of infected tissue with the antifungal drug amphotericin B (AmB). D. Treatment with proangiogenic growth factors VEGF and fibroblast growth factor (FGF) counteracts the action of gliotoxin and enhances the influx of polymorphonuclear leukocytes and antifungal drugs to the site of infection.

*M*. *tuberculosis*, the causative agent of tuberculosis, has not been found to produce bacterial angiogenic factors, yet its ability to survive and persist in the host is intimately related to pathological angiogenesis [6,19]. *M*. *tuberculosis* elicits the formation of dense cellular aggregates (granulomas) that wall off the pathogen. The presence of viable mycobacteria within macrophages in granulomas triggers VEGF-dependent tumor–like angiogenesis associated with dysfunctional (leaky) blood vessels. Dysregulated angiogenesis further limits perfusion of the granuloma core, exacerbating hypoxia and causing caseating necrosis, a hallmark of this infection (Fig 1). Pathological angiogenesis may be an important cause of inadequate delivery of antibiotic drugs and immune cells to the center of the granuloma, necessitating multidrug combinations and protracted treatment courses to eradicate the disease [6,19].

## Attenuation of Host Angiogenesis Creates Sequestered Niches Where Pathogens Persist

Inhibition of angiogenesis during infection interferes with tissue healing and facilitates a hypoxic and/or necrotic milieu that compromises immune function and favors pathogen persistence (Table 1). *A*. *fumigatus* produces life-threatening pulmonary infection in immunocompromised individuals, principally patients with hematological malignancies and recipients of hematopoietic stem cell transplantation [20]. In the setting of profound neutropenia, airborne spores (conidia) are inhaled into pulmonary alveoli, where they germinate and form tissue-invasive filaments (hyphae) that bore through the alveolar–capillary barrier and invade pulmonary arterioles [20]. Angioinvasion is associated with endothelial injury, tissue factor expression, triggering of the coagulation cascade, and platelet activation [21]. Collectively, these processes impair vascular perfusion of *Aspergillus*-infected lung tissue, producing a necrotic core where fungal hyphae proliferate abundantly, surrounded by a peripheral zone of alveolar hemorrhage (Fig 1) [22]. The importance of adaptation to hypoxia for *A*. *fumigatus* pathogenesis is underscored by work showing that deletion of the *SrbA* gene, which is essential for survival in hypoxic environments, renders *A*. *fumigatus* nonvirulent [23]. Invasive pulmonary aspergillosis is associated with a rapid increase in tumor necrosis factor (TNF)α transcription in mouse lungs but down-regulation of angiogenesis mediators that are normally induced by this cytokine: VEGF, FGF, and their receptors [24]. Uncoupling of inflammatory mediators and angiogenesis is further evident in reduced microvascular density around necrotic pulmonary lesions [7,25]. Inhibition of angiogenesis is mediated by *A*. *fumigatus* secondary metabolites, chiefly gliotoxin, under the transcriptional control of LaeA [24]. Attenuated angiogenesis likely perpetuates tissue hypoxia and limits trafficking of immune cells and antifungal drugs into the site of *Aspergillus* infection [5,20]. Thus, the vasculopathy of invasive aspergillosis plays a pathogenic role by restricting innate immune cell traffic to the site of infection and optimizing local growth conditions for the fungus.

## Modulation of Host Angiogenesis as a Therapeutic Target in Infections

The concept of angiogenesis modulation as a novel microbial virulence factor suggests the potential for attenuating pathogenicity using vascular-active molecules. Cancer research has produced numerous monoclonal antibodies and small molecules that target VEGF and its receptor (VEGFR) [3,26,27]. Originally thought to deprive tumors of their vascular supply, these agents are now believed to increase perfusion and alleviate hypoxia by normalizing tumor vasculature [27]. Similarly, angiogenesis modulators have little if any direct antimicrobial activity but act synergistically with conventional antimicrobials by enhancing drug delivery to the anatomical site of infection.

This idea has been explored in a rabbit model of *M*. *tuberculosis* infection and a zebrafish model of *Mycobacterium marinum* infection [6,19]. Inhibition of angiogenesis using bevacizumab, an anti-VEGF-A monoclonal antibody [6], and VEGFR tyrosine kinase inhibitors SU5416 and pazopanib [19] prevented the formation of abnormal ectopic blood vessels around mycobacterial granulomas, improved granuloma perfusion, and decreased necrotic tissue volume, bacterial burden, and dissemination without directly affecting mycobacterial growth in vitro (Fig 1) [6,19]. Moreover, pazopanib treatment alone significantly increased survival in the *M*. *marinum* zebrafish model, and SU5416 potentiated the activity of the first-line antituberculosis drug rifampicin [19].

In contrast, the vasculopathy of invasive aspergillosis is reversed following repletion of proangiogenic factors [7]. Treatment with VEGF and FGF alone significantly increased survival in a neutropenic mouse model of invasive pulmonary aspergillosis, and both growth factors acted synergistically with the antifungal drug amphotericin B to enhance survival and decrease pulmonary fungal burden (Fig 1) [7]. FGF enhanced the generation of CD31-positive vessels and was associated with neutrophil infiltrates around *A*. *fumigatus* infection sites. Interestingly, FGF had a more potent effect on mouse survival and fungal burden than did VEGF, a fact consistent with the association of VEGF with immature and hyperpermeable blood vessels [7].

These preliminary findings should be viewed within the context of the grand challenges to healthcare presented by *M*. *tuberculosis* and *A*. *fumigatus* [28,29]. *M*. *tuberculosis* infects one-third of the world’s population and is the second greatest cause of infectious mortality worldwide [29]. Currently, treatment involves complex multidrug regimens lasting months, which many patients do not tolerate. Moreover, extensive resistance to antituberculosis drugs has emerged in some parts of the world [29]. Invasive aspergillosis is lethal in about one-third of patients [30], and resistance to voriconazole, the foremost drug used to treat this infection, is spreading across Europe and Asia [31]. Vascular targeted therapies may herald the prospect of more effective antimicrobial drug delivery, allowing shorter, simpler treatment regimens and more efficient pathogen clearance.
