# Correction: Modulation of Host Angiogenesis as a Microbial Survival Strategy and Therapeutic Target

**DOI:** 10.1371/journal.ppat.1005838

**Published:** 2016-08-17

**Authors:** Nir Osherov, Ronen Ben-Ami

Reference 16 refers to the incorrect article. The correct reference is: Riess T, Andersson SG, Lupas A, Schaller M, Schafer A, et al. (2004) Bartonella adhesin a mediates a proangiogenic host cell response. J Exp Med 200: 1267–1278. doi: 10.1084/jem.20040500. pmid:15534369
